# Supplementary material for: Enhanced intelligent train operation algorithms for metro train based on expert system and deep reinforcement learning
Source: PLoS One. 2025 May 21;20(5):e0323478. doi: 10.1371/journal.pone.0323478 (PMC12094749; doi:10.1371/journal.pone.0323478)
Supplement: Attachment — Submitted filename: Response to Reviewers.docx [file pone.0323478.s001.docx]

**Responses to Reviewers’ Comments**

Dec. 2, 2024

**Dear Editorial Office of《PLOS ONE》:**

On behalf of my coauthors, we thank you very much for giving us an opportunity to revise our manuscript. We thank the chief editors, responsible editors and reviewers of 《**PLOS ONE**》for their constructive comments and suggestions on our manuscript entitled **“Enhanced Intelligent Train Operation Algorithms for Metro Train based on Expert System and Deep Reinforcement Learning”. (ID: PONE-D-24-17033** ), which have further improved the quality of our manuscript. We have studied reviewers’ comments carefully and made revision marked in red in the paper. We have tried our best to revise our manuscript according to the comments. Attached is the revised version, which we would like to submit for your kind consideration.

We would like to express our great appreciation to reviewers for comments on our paper. Looking forward to hearing from you.

Yours sincerely.

The authors: **Yuhhu Huang, Wenzhu Lai, Dewang Chen, Geng Lin, Jiateng Yin**

**Acknowledgement:**

We thank the reviewers and the editors for their careful reading of our manuscript: **“Enhanced Intelligent Train Operation Algorithms for Metro Train based on Expert System and Deep Reinforcement Learning”. (ID: PONE-D-24-17033** )**,** and for their useful comments and suggestions. Those comments are all valuable and very helpful for revising and improving our paper, as well as the important guiding significance to our researches. We have studied comments carefully and have made correction which we hope meet with approval. Revised portion are marked in red in the paper. The main corrections in the paper and the responds to the reviewer’s comments are as following:

Responds to the reviewer’s comments:

**List of Responses**

**Review Comments to the Author**

**To Reviewer #1:**

**1.What is the necessity of proposing two algorithms with the same functionality in a paper?**
**Response:** We are very grateful to the reviewers for your insightful comments on our manuscript.

The necessity of proposing both EITO_E_ and EITO_P_ lies in their complementary strengths. EITO_E_ leverages expert knowledge and heuristic rules to provide a robust baseline for intelligent train operation, while EITO_P_ uses deep reinforcement learning (PPO) to optimize multiple objectives dynamically. By presenting both algorithms, we aim to demonstrate how expert knowledge can be effectively integrated with advanced machine learning techniques to enhance train operation performance. This dual approach allows for a comprehensive evaluation of their effectiveness under varying conditions, showcasing the versatility and adaptability of our proposed solutions.

We have incorporated a response to the necessity of proposing two algorithms with identical functionality in the sixth paragraph from the end in the introduction section, as indicated in the red text of the Introduction.

**2. How do these 2 algorithms ensure punctuality**
**Response:** We are very grateful to the reviewers for your insightful comments on our manuscript.

Both EITO_E_ and EITO_P_ ensure punctuality through real-time adjustments based on current train conditions and speed limits. EITO_E_ uses expert rules to allocate trip times effectively, while EITO_P_ employs reinforcement learning to dynamically adjust acceleration and braking strategies. The algorithms continuously monitor the train's position and speed, allowing them to make timely decisions that keep the train on schedule. Specifically, the reward function in EITO_P_ penalizes deviations from planned trip times, reinforcing behaviors that promote punctuality.

The analysis in this section is as discussed in red in Section 3 "EITO Algorithm Design" of the article.

**3. Eq. 5 seems allow overspeed instead of forbidden overspeed**
**Response:** We are very grateful to the reviewers for your insightful comments on our manuscript. We appreciate your observation regarding Eq. (5). The intention of this equation is to ensure that the train's speed remains within the designated limits. We will clarify the equation in the manuscript to explicitly state that the evaluation index $I_{s}$is designed to enforce speed limits, and we will revise the language to prevent any misinterpretation. The condition should ensure that if the speed exceeds the limit, the index will reflect a violation, thereby discouraging overspeeding. As shown in the red text under Eq. (5).

**4. How to demonstrate the dynamic adjustment ability of EITOp? No clear indicators such as computational efficiency, computational complexity, and computational time were seen.**

**Response:** We are very grateful to the reviewers for your insightful comments on our manuscript. Thank you for highlighting this important aspect. Below is the analysis of how EITO_P_ demonstrates its dynamic adjustment capabilities,

Demonstration of Dynamic Adjustment Capabilities:

In Case 2, the flexibility of the EITO_P_ algorithm was showcased by simulating the same track segment under different planned travel times. EITO_P_ is capable of adjusting arrival times in response to real-time notifications (either advancing or delaying), thereby optimizing train operation strategies.

Adaptability:

In Case 3, EITO_P_ was tested in continuous station intervals with complex speed restrictions and gradients, proving its robustness under complex conditions. EITO_P_ maintains the safety and punctuality of train operations under varying speed limits and gradient conditions.

Metrics for Computational Efficiency, Complexity, and Time:

Online Learning Process:

In Case 1, during the online learning process of EITO_P_, energy consumption was reduced from 380 to 364 units after approximately 80 rounds of training, gradually approaching the optimal value. Meanwhile, the running time fluctuated between 100-102 seconds, meeting the requirement for on-time performance. This indicates that EITO_P_ can reduce energy consumption during the online learning process while adhering to the constraints of running time error.

Flexible Adjustment of Travel Time:

In Case 2, EITO_P_ demonstrated its adaptability under planned travel times of 95 seconds and 115 seconds. EITO_P_'s ability to adjust arrival times according to real-time notifications further illustrates the intelligence of the system.

Performance Comparison on Complex Continuous Lines:

In Case 3, the performance of EITO_P_ on continuous lines with complex speed restrictions and gradients was compared with EITO_E_. EITO_P_ outperformed EITO_E_ in terms of total travel time and total length, showing better punctuality and energy efficiency.

**To Reviewer #2:**

**1. The questions or limitations in research should be made clear. Just saying ‘not sufficiently intelligent, self-learning and generalized’ is too general and not focused.
Response:** We are very grateful to the reviewers for your insightful comments on our manuscript. We appreciate your feedback and revised the manuscript to clearly articulate the specific limitations of current ATO systems in the first paragraph of the Introduction section of the red text.

While ATO systems have been increasingly embraced by many metro systems over the past decades due to their low cost and practicality, it is evident that they fall short in several critical areas. Firstly, the intelligence of these systems is limited; they often rely on predefined operational strategies and lack the dynamic adaptability to respond effectively to complex and unforeseen circumstances. Secondly, the absence of self-learning capabilities restricts their potential to improve efficiency and safety over time through the accumulation and analysis of operational data. Lastly, the generalization of these systems is constrained; they are typically tailored to specific lines and struggle to adapt to diverse line conditions, such as varying speed limits, gradients, and traffic flows, which limits their broader application. These limitations underscore the need for more advanced, intelligent, and adaptable train operation systems that can enhance operational efficiency, safety, and passenger comfort.

**2. The contributions should be only ‘contributions’, and the works done are not contributions.**
**Response:** We are very grateful to the reviewers for your insightful comments on our manuscript. Here is a refined and simplified presentation of the contributions, focusing solely on the 'contributions' aspect as per the reviewer's request:

1. Integration of Expert Knowledge with Deep Reinforcement Learning: We introduce a novel approach that integrates expert system-based rules, distilled from experienced drivers, with the Proximal Policy Optimization (PPO) algorithm. This integration results in the development of EITO_E_ and EITO_P_ algorithms, which not only provide a robust operational baseline for intelligent train operation but also dynamically optimize multiple objectives, enhancing the adaptability and efficiency of train control systems.

2. Development of EITO_E_ Algorithm: The EITO_E_ algorithm is developed by encapsulating heuristic rules and inference methods from expert drivers within an expert system framework. This innovation allows for the generation of control strategies independent of offline speed profiles, thereby offering a flexible and adaptive operational approach that is responsive to real-time train operation requirements.

3. EITO_P_ Algorithm for Multi-Objective Optimization: Extending the capabilities of EITO_E_, the EITO_P_ algorithm utilizes PPO to optimize key operational objectives including safety, punctuality, energy efficiency, and passenger comfort. A significant contribution of EITO_P_ is its real-time adjustment of acceleration and braking strategies based on current train conditions and speed limits, which is crucial for maintaining energy efficiency and punctuality in metro train operations.

**3.There are too many references in the methodology part, for example, the train motion model was referred to reference 23, train acceleration system to reference 25, indices of model evaluation are usually evaluation from five aspects.**

**Response:** We are very grateful to the reviewers for your insightful comments on our manuscript. We appreciate your observation regarding the references in the methodology section.

We have carefully reviewed and reduced the excessive references in the methodology section that were not essential, focusing on the most relevant studies that directly support our methodology to alleviate the reader's burden and enhance the coherence of the paper. The citations we have reduced in the methodology are not necessary for understanding the content, or can be expressed more clearly without references. We have reduced or simplified these citations (such as the original references [24], [25], [26], [27], [28]). This will enhance clarity and readability while ensuring that the foundational concepts are adequately covered.

Thank you again for your insightful suggestions.

**4.The methodology part should highlight the contribution of this work.**
**Response:** Thank you for your valuable feedback. We have carefully revised the methodology section to more clearly highlight the contributions of this work. Below is the updated description that emphasizes our novel approach and its significance:

Clarity of Innovation:

Our study introduces a novel approach by integrating expert knowledge with advanced machine learning techniques, specifically the Proximal Policy Optimization (PPO) algorithm, to develop two enhanced intelligent train operation algorithms: EITO_E_ and EITO_P_. These algorithms represent a significant departure from traditional methods, offering a more intelligent and adaptive control strategy for metro train operations.

Detailed Method Description:

We have elaborated on the design and implementation of the EITO_E_ and EITO_P_ algorithms, detailing how they leverage an expert system to generate control strategies without reliance on predefined speed profiles. The EITO_E_ algorithm encapsulates heuristic rules and inference methods derived from expert drivers, while EITO_P_ employs PPO to dynamically optimize multiple operational objectives such as safety, punctuality, energy efficiency, and passenger comfort.

Emphasis on Methodological Advantages:

We underscore the advantages of our methodologies, which include improved efficiency, adaptability, safety, and comfort. Our algorithms demonstrate superior performance in complex conditions, such as varying speed limits and gradients, showcasing their robustness and practical applicability in real-world scenarios.

Demonstration of Method Applicability:

The applicability of our methods across different operational conditions, including diverse trip times, speed restrictions, and gradient conditions, is demonstrated through rigorous testing. This versatility is crucial for the practical implementation of intelligent train operation algorithms in urban rail transit systems.

Comparative Analysis:

Through comparative analysis with existing methods, we have showcased how our algorithms provide superior solutions, particularly in terms of energy conservation and passenger comfort. The results indicate that EITO_E_ and EITO_P_ outperform current intelligent driving algorithms and manual driving in these critical areas.

Significance of Results:

Finally, we discuss the theoretical and practical implications of our findings for the field of intelligent transportation systems. Our research not only contributes to the theoretical development of intelligent train operation but also lays a foundation for future innovations in this domain.

This refined summary encapsulates the key contributions of our work and aligns with the high standards expected by the reviewer, ensuring that the innovative aspects of our research are clearly articulated and understood.

**5. Section 2 Problem formulation is confusing. Why 2.3 problem statement appears at the end of this section?**
**Response:** We are very grateful to the reviewers for your insightful comments on our manuscript. Thank you for pointing this out. We reorganized Section 2 to improve the flow and clarity and Logic of the problem formulation.

The original title "2. Problem Formulation" is changed to "2. Problem Formulation and Control Model" to better reflect the content.

**1). Reorganization of Section 2 Structure:**

To enhance clarity, Section 2 is divided into two distinct subsections: Problem Statement and Control Model. This division aids readers in understanding the purpose and content of each part more effectively.

Modifications are as follows:

2.1 Problem Statement: The existing Problem Statement, previously section 2.3, is moved to section 2.1, beginning with a clear statement of the research objectives and challenges.

2.2 Control Model of Train: The original control model section, including mathematical models and performance indicators, is placed in section 2.2, focusing on introducing the methods and models used to address the problem.

**2). Clarifying the Purpose and Content of Each Section:**

A brief introduction is added at the beginning of each subsection to explain its purpose and content, helping readers comprehend the significance and role of each part.

Specific modifications include:

At the beginning of section 2.1, add: "This section will clearly articulate the problems that this study aims to address."

At the beginning of section 2.2, add: "Based on the problem statement, this section will provide a detailed introduction to the control models, mathematical methods used to achieve these objectives, and define the relevant performance metrics."

**3). Ensuring Logical Coherence:**

The logical relationships between the parts of Section 2 are reviewed to ensure a smooth transition from the problem statement to the control model, with each part being closely related.

Specific modifications include:

At the end of section 2.1, add a transitional sentence, such as: "Based on the problem statement outlined above, the next section will provide a detailed introduction to the specific control models and methods used to achieve these objectives."

Ensure that the content of section 2.2 is directly related to the problems and objectives presented in section 2.1.

**6. The Conclusion section should be improved by give more conclusions in the second paragraph of this section. What can be learned from the research and results of this work? Currently, it is too simple and general.**
**Response:** We appreciate your suggestion for enhancing the conclusion. We will expand the second paragraph to summarize the key findings of our research, emphasizing the implications of our results for future metro train operations and the potential for further research in this area. This will provide a more comprehensive wrap-up of our study.

In this study, we introduced two Enhanced Intelligent Train Operation (EITO) algorithms designed for continuous metro operation control tasks, showcasing their ability to operate without the need for tracking offline speed profiles or relying on exact train model information. Our approach leverages an expert system to generate EITO_E_ outputs based on driver experience and redefines key elements of the Proximal Policy Optimization (PPO) algorithm to develop EITO_P_, which optimizes multiple operational objectives online. Through comparative analysis with existing intelligent driving algorithms and manual driving data, we demonstrated the superiority of our proposed algorithms in terms of safety, punctuality, energy efficiency, and passenger comfort.

From the research and results of this work, several key conclusions can be drawn:

Flexible Intelligent Control: The EITO algorithms demonstrate the viability of intelligent train operations adaptable to real-time conditions, enhancing efficiency and adaptability beyond traditional, preset profiles.

Expert System Collaboration: Integrating expert system insights with EITO_E_ significantly bolsters performance, underscoring the collaboration between human expertise and machine learning in intelligent train operations.

Multi-Objective Optimization: EITO_P_, leveraging PPO, distinguishes itself by dynamically juggling safety, punctuality, energy efficiency, and comfort—often competing objectives—in a holistic control strategy.

Robustness in Complexity: Both EITO_E_ and EITO_P_ showcase robust performance in complex operational environments, with EITO_P_ particularly adept at adjusting to varying trip times, crucial for real-world operational efficiency.

Energy and Comfort Superiority: Our algorithms surpass current methods in energy conservation and passenger comfort, tackling critical urban rail transit challenges and validating their practical application.

While our algorithms are promising, future work will focus on enhancing EITO_P_'s dynamic adjustment capabilities and exploring cooperative control strategies for energy savings, including optimizing train schedules for regenerative energy utilization.

**7.There are many format mistakes. For instance, Fig. 3 is inserted in the middle of the paragraph.**

**Response:** Thank you for your attention to detail. We have inserted the Fig. 3 in the proper place.

Additionally, we have conducted a thorough review of the manuscript to correct any formatting issues, including the placement of figures and tables. Ensuring proper formatting is essential for clarity and professionalism, and we appreciate your guidance in this matter.

We appreciate for Editors/Reviewers’ warm work earnestly, and hope that the correction will meet with approval.

Once again, thank you very much for your comments and suggestions.
