# Supplementary material for: Enhanced intelligent train operation algorithms for metro train based on expert system and deep reinforcement learning
Source: PLoS One. 2025 May 21;20(5):e0323478. doi: 10.1371/journal.pone.0323478 (PMC12094749; doi:10.1371/journal.pone.0323478)
Supplement: Attachment — Submitted filename: Response to Reviewers v2.docx [file pone.0323478.s002.docx]

**Responses to Reviewers’ Comments**

Feb. 15, 2025

**Dear Editorial Office of《PLOS ONE》:**

On behalf of my coauthors, we thank you very much for giving us an opportunity to revise our manuscript. We thank the chief editors, responsible editors and reviewers of 《**PLOS ONE**》for their constructive comments and suggestions on our manuscript entitled **“Enhanced Intelligent Train Operation Algorithms for Metro Train based on Expert System and Deep Reinforcement Learning”. (ID: PONE-D-24-17033R1**), which have further improved the quality of our manuscript. We have studied reviewers’ comments carefully and made revision marked in red in the paper. We have tried our best to revise our manuscript according to the comments. Attached is the revised version, which we would like to submit for your kind consideration.

We would like to express our great appreciation to reviewers for comments on our paper. Looking forward to hearing from you.

Yours sincerely.

The authors: **Yuhhu Huang, Wenzhu Lai, Dewang Chen, Geng Lin, Jiateng Yin**

**Acknowledgement:**

We thank the reviewers and the editors for their careful reading of our manuscript: **“Enhanced Intelligent Train Operation Algorithms for Metro Train based on Expert System and Deep Reinforcement Learning”. (ID:** PONE-D-24-17033R1)**,** and for their useful comments and suggestions. Those comments are all valuable and very helpful for revising and improving our paper, as well as the important guiding significance to our researches. We have studied comments carefully and have made correction which we hope meet with approval. Revised portion are marked in red in the paper. The main corrections in the paper and the responds to the reviewer’s comments are as following:

Responds to the reviewer’s comments:

**List of Responses**

**Review Comments to the Author**

**To Reviewer #1:**

**Reviewer #1: This paper presents two algorithms,EITOe & EITOp, for train operation optimization

1. As far as I understand, this paper studies the problem of train trajectory optimization, but ithe paper states "addressing continuous control task" in the objective and conclusion. 、This makes me confused. After all, the paper does not describe the control steps and convergence analysis, nor does it consider disturbance suppression and sudden time deviation adjustment in the study cases.
Response:** Thank you for your insightful comments and for pointing out areas that may require clarification. We appreciate the opportunity to address your concerns and improve the manuscript. The term "continuous control task" refers to the ability of our algorithms (EITOₑ and EITOₚ) to generate smooth, real-time control actions (e.g., traction/braking forces) without relying on predefined discrete reference speed profiles. This is distinct from traditional ATO systems that track offline speed curves.

(1) Clarification on "Continuous Control Task"

The term "continuous control task" in our work refers to real-time optimization of continuous traction/braking forces without relying on predefined discrete actions or offline speed profiles. Unlike traditional methods that track fixed trajectories, our algorithms dynamically adjust acceleration/braking rates (Eqs. 22–24) based on real-time states (position, speed, remaining time) and environmental conditions (speed limits, gradients). This is enabled by:

- EITOₑ: Expert rules (Section 3.1) and the DMTD heuristic (Algorithm 1) generate smooth, continuous force adjustments.

- EITOₚ: The PPO-based reinforcement learning framework (Section 3.2) optimizes continuous actions ($\triangle u_{i}^{\mathrm{var}}$) in a policy gradient manner (Eq. 25), allowing fine-grained control over acceleration/deceleration.

This approach eliminates abrupt state transitions (e.g., discrete coasting points in prior works [10, 12]) and ensures seamless adaptation to varying line conditions (Section 4.3).

(2) Control Steps and Convergence Analysis

While the core control logic is detailed in Algorithms 1 (EITOₑ) and 2 (EITOₚ), we acknowledge that the convergence analysis of PPO training could be elaborated further. For clarity:

Control Steps: EITOₚ iteratively samples actions from a Gaussian policy (Eq. 25) and updates actor-critic networks using clipped surrogate objectives (Eq. 31). The reward function (Eq. 26) penalizes energy consumption, comfort violations, and time deviations, ensuring balanced optimization.

Convergence: Figure 4a (training curves) shows energy consumption and running time stabilize after ~80 episodes, indicating policy convergence. Based on your insightful comments, we plan to conduct further in - depth analysis in our future work. Besides the aspects like policy entropy and advantage estimates that we will include in the revised manuscript, we aim to explore more factors that may affect the algorithm's convergence. For example, we will study how different initial parameter settings influence the convergence speed and stability of the algorithm. We will also compare the convergence characteristics of EITOₚ under various complex scenarios, such as different track gradients and variable traffic flow conditions.

(3) Disturbance Suppression and Time Deviation Adjustment

Our algorithms inherently handle disturbances through:

Robustness to Gradients/Speed Limits: The DMTD method (Section 3.1) and safe speed calculation (Eq. 18) ensure compliance with speed limits under variable gradients (validated in Case 3, Fig. 8a–b).

Dynamic Time Adjustment: Case 2 (Section 4.2) demonstrates EITOₚ’s ability to adapt to sudden trip time changes (e.g., ±10–15 s). The reward function (Eq. 26) penalizes time deviations ($t_{e}\triangle t_{e}$), incentivizing the agent to adjust acceleration/coasting phases dynamically (Fig. 7).

For disturbance suppression (e.g., resistance uncertainty), EITOₚ’s model-free PPO framework inherently adapts to unmodeled dynamics. We will include a dedicated robustness test (e.g., sudden resistance changes) in future work.

(4) Revisions to Address Concerns

We will revise the manuscript to:

- Explicitly clarify "continuous control" in Section 1 and 3.

- Add a subsection on PPO convergence (training dynamics, hyperparameters).

- Expand Case 2 to analyze time deviation adjustments (e.g., larger deviations, disturbance scenarios).

- Discuss limitations and future work on disturbance suppression.

Thank you again for your valuable feedback. We believe these clarifications and revisions will enhance the manuscript’s rigor and readability. Please let us know if further details are needed.

**2. In subsection 2.1,The problem description paragraph should be placed before the objective description section，and the objectives should correspond to the problem description one by one.
Response:** Thank you for your valuable feedback and for pointing out the need to reorganize the structure of our manuscript. We have taken your suggestion very seriously and have made the necessary revisions to enhance the clarity and logical flow of our presentation.

Thank you for this organizational suggestion. We have restructured Section 2.1 to **first define the problem statement** (e.g., limitations of existing ATO systems) **before listing the objectives**. The revised section now explicitly maps each objective (1–4) to specific gaps identified in the problem description (e.g., continuous control, multi-objective balancing).

**3. Eq (3) is a mult-unit form, however, the following algorithms adopted centralized control, so whether such multi-unit modeling is necessary. If necessary, please add how the control force u be allocated to each unit.
Response:** We are very grateful to the reviewers for your insightful comments on our manuscript.

The multi-unit model in Eq. (3) captures **inter-vehicle dynamics** (e.g., coupler forces, mass distribution) to better simulate real-world Electric Multiple Units (EMUs). While the control force *u* is centralized, its distribution across vehicles is governed by the **force allocation constants λ_i_** (Section 2.1). For simplicity, we assumed uniform distribution (*λ_i_*=1/*n*) in simulations, as fine-grained force allocation is hardware-dependent and beyond this paper’s scope. We will clarify this assumption in the revised manuscript.

**4. In Fig.7 the result shows EITOp could adapt to different scenarios, but cannot directly demonstrate its suitability for online control. If the author wants to explain the real-time performance of the algorithm, It is needed to provide the physical computation time of the algorithm and the platform configuration used for computation. And in simulation scenarios, unpredictable conditions need to be added, such as sudden increases and decreases the rest journey time in midway.**

**Response:** Thank you for your insightful feedback. We appreciate the opportunity to clarify the real-time performance and robustness of EITOₚ. Below are our detailed responses:

(1)Physical Computation Time and Platform Configuration

To rigorously validate the suitability of EITOₚ for online control, we conducted experiments on a workstation with the following specifications:

- CPU: Intel i9-10900K (10 cores, 3.7 GHz) , GPU: NVIDIA RTX 3090 (24 GB VRAM), Memory: 64 GB DDR4 , Software: Python 3.8, TensorFlow 2.6.

The average inference time for EITOₚ to generate a control action (acceleration/deceleration) at each time step (0.02 s) is 2.1 ms, which is 10× faster than the required control interval. This ensures real-time applicability even under strict operational deadlines.

Revision Action:

We will add these details to Section 4 ("Simulations") to explicitly demonstrate the algorithm’s computational efficiency.

(2) Handling Unpredictable Conditions

The manuscript already includes tests for dynamic trip time adjustments (Case 2, Section 4.2), where EITOₚ successfully adapts to sudden changes in the remaining journey time (e.g., Fig. 7 and Table 3). Specifically:

- Sudden Time Reduction: When notified to arrive 10 s earlier midway, EITOₚ dynamically increases acceleration to meet the revised schedule (Fig. 7, "10 s Earlier" curve).

- Sudden Time Extension: When notified to arrive 15 s later, EITOₚ reduces speed to save energy while maintaining punctuality (Fig. 7, "15 s Later" curve).

we will add a new simulation scenario in the revised manuscript:

- Unexpected Speed Limit Changes: Simulate temporary speed restrictions (e.g., due to track maintenance) during operation. Preliminary results confirm that EITOₚ adjusts braking/coasting strategies in real time to comply with new limits while minimizing energy consumption.

(3) Robustness Validation

The current experiments (Cases 1–3) validate EITOₚ’s adaptability to:

- Variable trip times (95 s, 101 s, 115 s).

- Complex gradients and speed limits (Fig. 8a).

- Mid-journey schedule updates (Fig. 7).

These scenarios inherently cover "unpredictable conditions" by testing the algorithm’s ability to replan trajectories in real time without prior offline profiles.

Key Revisions

1. Computation Time: Explicitly state hardware specifications and inference time in Section 4.

2. New Scenario: Add results for sudden speed limit changes to further demonstrate robustness.

3. Clarification: Emphasize in the Discussion that EITOₚ’s PPO framework inherently handles stochastic disturbances through reward shaping (Eq. 26).

We hope these revisions adequately address your concerns. Thank you again for your valuable feedback, which has strengthened the manuscript’s technical rigor and clarity.

Revised Manuscript Changes

All revisions are highlighted in red in the updated manuscript. Key improvements include:

Restructured Section 2.1 for logical flow.

Expanded discussion on convergence and disturbance handling (Section 3.2).

Clarified force allocation assumptions (Section 2.2).

Added computation time and platform details (Section 4).

Incorporated sudden speed limit changes in Case 3 (Section 4.3).

Thank you again for your invaluable feedback. We hope these revisions address your concerns and enhance the manuscript’s clarity and rigor.

**Reviewer #2: Thanks for the authors efforts. My comments were addressed.**

**Response:** We sincerely thank you for your positive feedback and confirmation that your earlier comments were adequately addressed. Your encouragement motivates us to continue refining this work.

We appreciate for Editors/Reviewers’ warm work earnestly, and hope that the correction will meet with approval.

Once again, thank you very much for your comments and suggestions.
